# Dual-Layer Spectral CT for Locoregional Assessment of Rectal Cancer: A Comparison with MRI in a Pilot Study

**DOI:** 10.3390/diagnostics16182948

**Published:** 2026-09-12

**Authors:** Silvio Mazziotti, Giorgio Ascenti, Tommaso D’Angelo, Ludovica R. M. Lanzafame, Pasquale Arena, Alfredo Blandino, Sarah Doria, Simone Barbera, Robert Runinski, Velio Ascenti, Laura Pipino, Giuseppe Costantino, Anna Viola, Giuseppe Cicero

**Affiliations:** 1 Diagnostic and Interventional Radiology Unit, BIOMORF Department, University Hospital “Policlinico G. Martino”, Via Consolare Valeria 1, 98125 Messina, Italy; 2 Radiology Department, Fondazione IRCCS Cà Granda Ospedale Maggiore Policlinico, 20122 Milan, Italy; 3 IBD-Unit, Department of Clinical and Experimental Medicine, University of Messina, Policlinico, Pad B, Via Consolare Valeria, 1, 98125 Messina, Italy

**Keywords:** rectal cancer, dual-layer spectral CT, locoregional staging, monoenergetic imaging, magnetic resonance

## Abstract

**Background/Objectives:** To evaluate the concordance of dual-layer spectral CT (DLSCT) with MRI in locoregional staging of rectal cancer, focusing on 40 keV virtual monoenergetic reconstructions. **Methods:** This retrospective single-center study included 31 patients (mean age, 69 ± 13.3 years) with rectal cancer who underwent both MRI and DLSCT within 30 days before treatment. Venous-phase CT images were reconstructed at 40 keV. Assessed parameters included tumor location, longitudinal extent, distance from the anorectal junction and anal verge, mesorectal infiltration, circumferential resection margin (CRM) involvement, suspicious lymph nodes, extramural vascular invasion (EMVI), desmoplastic reaction, peritoneal involvement, and anal canal invasion. Two radiologists performed consensus readings. Paired continuous measurements were compared with the Wilcoxon signed-rank test. Agreement for categorical variables was evaluated using Cohen’s kappa; sensitivity, specificity, and accuracy were calculated using MRI as the imaging reference standard. **Results:** No significant differences were found between MRI and DLSCT for the continuous measurements evaluated. Complete agreement was observed for CRM involvement, anal canal invasion, peritoneal involvement, desmoplastic reaction, and suspicious lymph-node classification (accuracy, 100%; κ = 1.00). Agreement was almost perfect for mesorectal infiltration (κ = 0.84). For EMVI, DLSCT reproduced all MRI-positive classifications but generated two additional positive findings, resulting in 100% sensitivity, 71.4% specificity, 93.5% accuracy, and substantial agreement (κ = 0.79). **Conclusions:** DLSCT showed high concordance with MRI for several locoregional imaging features of rectal cancer. It may provide complementary information, particularly when MRI is contraindicated or inconclusive. In addition, when DLSCT is performed for systemic staging, it has the potential to provide both locoregional and distant disease assessment within a single examination. However, these findings do not establish equivalence to MRI or diagnostic accuracy against histopathology, and further prospective studies with pathological correlation are warranted.

## 1. Introduction

Colorectal cancer is the third most commonly diagnosed malignancy worldwide, with rectal cancer accounting for approximately one-third of cases [1,2]. Diagnosis and treatment rely on a multimodal approach, where cross-sectional imaging plays a pivotal role in appropriate staging, treatment planning, and prognostic stratification [3,4].

The clinical management of locally advanced rectal cancer has undergone a significant paradigm shift in recent years, particularly with the widespread adoption of Total Neoadjuvant Therapy (TNT) and “watch-and-wait” organ-preservation strategies for patients achieving a complete clinical response [5]. In this modern therapeutic landscape, the need for accurate baseline locoregional staging has become increasingly important. Identifying adverse imaging features, such as a threatened or involved mesorectal fascia and extramural vascular invasion, contributes substantially to multidisciplinary treatment selection, including decisions regarding upfront surgery, chemoradiotherapy, and total neoadjuvant therapy. Consequently, any imaging modality proposed for rectal cancer staging must not only detect the primary mass but must also exhibit the spatial and contrast resolution necessary to map the intricate microanatomy of the mesorectum. This pressing clinical need underscores the urgency of exploring advanced tomographic techniques that can mitigate the known logistical and technical limitations of conventional MRI, while maintaining the same rigorous standards of anatomical detail required by modern multidisciplinary tumor boards.

Currently, magnetic resonance imaging (MRI) is regarded as the reference standard for the locoregional staging of rectal cancer. High-resolution MRI provides clinically actionable information regarding the depth of tumor invasion, mesorectal fascia involvement, circumferential resection margin (CRM), extramural vascular invasion (EMVI), and regional nodal status [6,7,8,9]. Nevertheless, its performance is not uniform across all staging tasks and depends on appropriate acquisition planes, image quality, and reader expertise. In particular, differentiation between tumors confined to the muscular wall and those showing minimal extramural extension may be challenging in the presence of desmoplastic or fibrotic changes [6,7,8,10], while nodal characterization remains imperfect because morphological and dimensional criteria cannot reliably distinguish all metastatic from benign mesorectal nodes [9,11]. In addition, MRI requires a dedicated examination and may be difficult or unsuitable in selected patients, including those with severe claustrophobia or non-MR-compatible implanted devices. These limitations provide a rationale for investigating complementary imaging approaches while maintaining MRI as the established reference modality for locoregional staging.

Endoscopic ultrasonography (EUS) also has an established role in the local assessment of rectal cancer, particularly for evaluating mural invasion in early-stage tumors and for distinguishing T1 from T2 lesions [7]. Its high spatial resolution allows detailed assessment of the rectal wall. In the study by Kocaman et al., using surgical pathology as the reference standard, EUS showed favorable T-staging performance compared with MDCT and MRI, whereas no significant difference among the modalities was observed for nodal staging [12]. Nevertheless, EUS has a limited field of view and does not provide the comprehensive assessment of the mesorectum and distant disease achievable with cross-sectional imaging.

Conventional computed tomography (CT) plays a complementary role and is primarily used for the assessment of distant metastatic disease. Although CT provides high spatial resolution, its relatively limited soft-tissue contrast has traditionally restricted its utility for local staging [3,4]. Recently, dual-energy computed tomography (DECT), and in particular dual-layer spectral CT (DLSCT), has emerged as a modality capable of overcoming these limitations [13,14,15]. Unlike source-based DECT systems, DLSCT simultaneously separates low- and high-energy photons, providing co-registered datasets that enable the reconstruction of virtual monochromatic images and material-specific maps. This intrinsic spectral separation reduces artifacts and improves lesion conspicuity, contrast resolution, and material differentiation [16,17].

Recent studies have further investigated the potential role of spectral CT in the locoregional assessment of colorectal and rectal cancer. Jia et al. compared DLSCT with high-resolution MRI for preoperative T-staging of early rectal adenocarcinoma and reported that 40-keV virtual monoenergetic images provided the most favorable image-quality metrics, with no significant difference in overall T-staging accuracy between DLSCT and MRI [18]. More recently, Chen et al. showed that low-keV DLSCT virtual monoenergetic images combined with multiplanar reformations improved preoperative colorectal cancer T-staging compared with conventional polyenergetic images, using histopathology as the reference standard [19]. In addition, a recent study comparing spectral detector CT-derived 40-keV virtual monoenergetic images, iodine density maps, conventional CT, and MRI for rectal cancer EMVI assessment found comparable diagnostic performance among the spectral reconstructions and MRI when evaluated against histopathology [20]. Together, these findings support the continued investigation of spectral CT as a complementary approach to established imaging techniques for locoregional assessment.

Low-keV virtual monoenergetic reconstructions may enhance iodine-related contrast and improve rectal tumor conspicuity. The purpose of this preliminary proof-of-concept study was to evaluate the concordance of DLSCT, with specific emphasis on 40-keV virtual monoenergetic reconstructions, with MRI for selected locoregional imaging features of rectal cancer.

## 2. Materials and Methods

### 2.1. Study Population

This retrospective, single-center study included 31 patients with histologically confirmed rectal cancer who met the eligibility criteria during the study period. Eligibility required the availability of both pretreatment magnetic resonance imaging (MRI), performed for locoregional staging, and dual-layer spectral CT (DLSCT), performed for distant disease assessment, with a maximum interval of 30 days between the two examinations. All examinations were performed between January 2023 and December 2025, before any surgical and/or radiotherapy treatment (Table 1).

### 2.2. CT Protocol

All CT examinations were performed with a dual-layer spectral CT scanner (IQon Spectral CT, Philips Healthcare, Best, The Netherlands). Acquisition was carried out at 120 kVp with automated tube current modulation. The gantry rotation time was 0.4 s, and collimation was set to 64 × 0.625 mm. A bolus-tracking technique was employed, with the region of interest placed within the abdominal aorta and a threshold of 100 Hounsfield units to trigger scanning. The multiphasic protocol included an arterial phase of the upper abdomen, a venous phase covering the entire abdomen, and a delayed phase limited to the lower abdomen.

Intravenous contrast medium was administered using a dual-syringe power injector. Iodinated contrast (Iomeprol, Iomeron^®^ 400 mgI/mL; Bracco Imaging, Milan, Italy) was injected at a dose of 1.5 mL/kg of body weight, followed by a 40-mL saline flush, both delivered at a flow rate of 3 mL/s through an antecubital vein.

For the purpose of this study, 40-keV virtual monoenergetic images were retrospectively reconstructed from the venous phase using dedicated spectral post-processing software (IntelliSpace Portal, version 11.1; Philips Healthcare). The 40-keV energy level was selected because low-keV virtual monoenergetic imaging increases iodine attenuation and may improve lesion-to-background contrast, while previous DLSCT studies in rectal cancer have reported favorable tumor conspicuity and image-quality metrics at this energy level [18]. The venous phase was selected because it is routinely included in oncologic CT protocols and provides relatively homogeneous enhancement of the rectal wall, mesorectum, adjacent pelvic organs, and regional lymph nodes, while allowing locoregional assessment to be performed on the same acquisition used for systemic staging. The acquisition parameters, including 120 kVp with automated tube current modulation and thin-section collimation (64 × 0.625 mm), reflected the clinical spectral CT protocol used for these examinations; thin collimation also permitted high-resolution multiplanar reconstructions. Low-keV imaging may increase iodine-related contrast but can also modify image-noise characteristics; therefore, window width and level were adjusted interactively according to reader preference during image interpretation.

### 2.3. MRI Protocol

All MRI examinations were performed on a 1.5-T scanner (Achieva, Philips Healthcare, Best, The Netherlands) equipped with a maximum gradient amplitude of 33 mT/m and a 16-channel phased-array surface coil. Patients were examined in the supine position without prior bowel preparation or rectal filling.

The protocol included high-resolution T2-weighted fast spin-echo sequences with a slice thickness of 3 mm, acquired in three planes: sagittal, axial, and coronal. For optimal tumor assessment, axial images were obtained perpendicular to the rectal lumen at the level of the lesion, while coronal images were acquired parallel to the rectal axis. In addition, diffusion-weighted imaging was performed using b-values of 0, 400, and 800 s/mm^2^.

To complement the high-resolution sequences, a wider-field T2-weighted fast spin-echo acquisition without fat suppression was obtained in the axial plane, extending from the aortic bifurcation to the anal sphincter. This sequence provided an overview of the pelvis and allowed assessment of more distant lymph node chains, including inferior mesenteric, lateral pelvic, and inguinal stations.

### 2.4. Image Review and Analysis

MRI and CT datasets were reviewed jointly by an experienced radiologist in oncologic imaging and a radiology resident, and all assessments were recorded by consensus. To minimize recall bias, CT and MRI examinations were interpreted in separate reading sessions performed at least six weeks apart. During each session, the readers were blinded to the findings of the other imaging modality, and the datasets were presented in randomized order.

For each case, a structured analysis was performed, including the assessment of tumor location within the rectum, its longitudinal extent, and the distance from both the anorectal junction and the anal verge. Depth of infiltration into the mesorectum and the relationship to the circumferential resection margin (CRM) were documented. In addition, suspicious lymph nodes were recorded, as well as signs of extramural vascular invasion (EMVI) and peritumoral desmoplastic reaction. Possible peritoneal involvement and direct extension to the anal canal were also systematically evaluated. All assessments were conducted using both dual-layer spectral CT (DLSCT) and magnetic resonance imaging (MRI).

### 2.5. Statistical Analysis

Statistical analyses were performed using MedCalc software (version 20; MedCalc Software Ltd., Ostend, Belgium). Data distribution was assessed using the Shapiro–Wilk test. Categorical variables were expressed as absolute numbers and percentages. Continuous variables were reported as means ± standard deviation (SD) when normally distributed and as medians with interquartile ranges (IQR) when non-normally distributed.

Paired continuous measurements obtained with MRI and DLSCT were compared using the Wilcoxon signed-rank test. For this analysis, a *p*-value < 0.05 was considered statistically significant. Because all examinations were interpreted by consensus, inter-reader agreement was not assessed.

Agreement between MRI and DLSCT classifications for categorical variables was evaluated using Cohen’s kappa coefficient. Kappa values were interpreted as follows: <0.20, slight agreement; 0.21–0.40, fair agreement; 0.41–0.60, moderate agreement; 0.61–0.80, substantial agreement; and 0.81–1.00, almost perfect agreement. MRI findings were used as the imaging reference standard for the comparative analysis. Sensitivity, specificity, and overall accuracy of DLSCT relative to MRI were calculated from 2 × 2 contingency tables, with 95% confidence intervals reported for sensitivity and specificity. These measures reflect concordance with MRI classifications and should not be interpreted as estimates of diagnostic accuracy against histopathology.

Given the retrospective and exploratory proof-of-concept design, no a priori sample-size calculation was performed. The study size was determined by the fixed number of eligible patients for whom both pretreatment MRI and DLSCT were available during the study period. Because no primary endpoint, clinically meaningful effect size, or target level of agreement had been prespecified for sample-size determination, an observed post-hoc power calculation was not performed. Instead, the precision of the estimates is conveyed by the reported confidence intervals, which also reflect the uncertainty associated with the limited sample size. The study was not designed or powered to demonstrate equivalence or non-inferiority between the two imaging modalities; therefore, the findings should be interpreted as preliminary estimates of concordance.

## 3. Results

**Figure 1:**
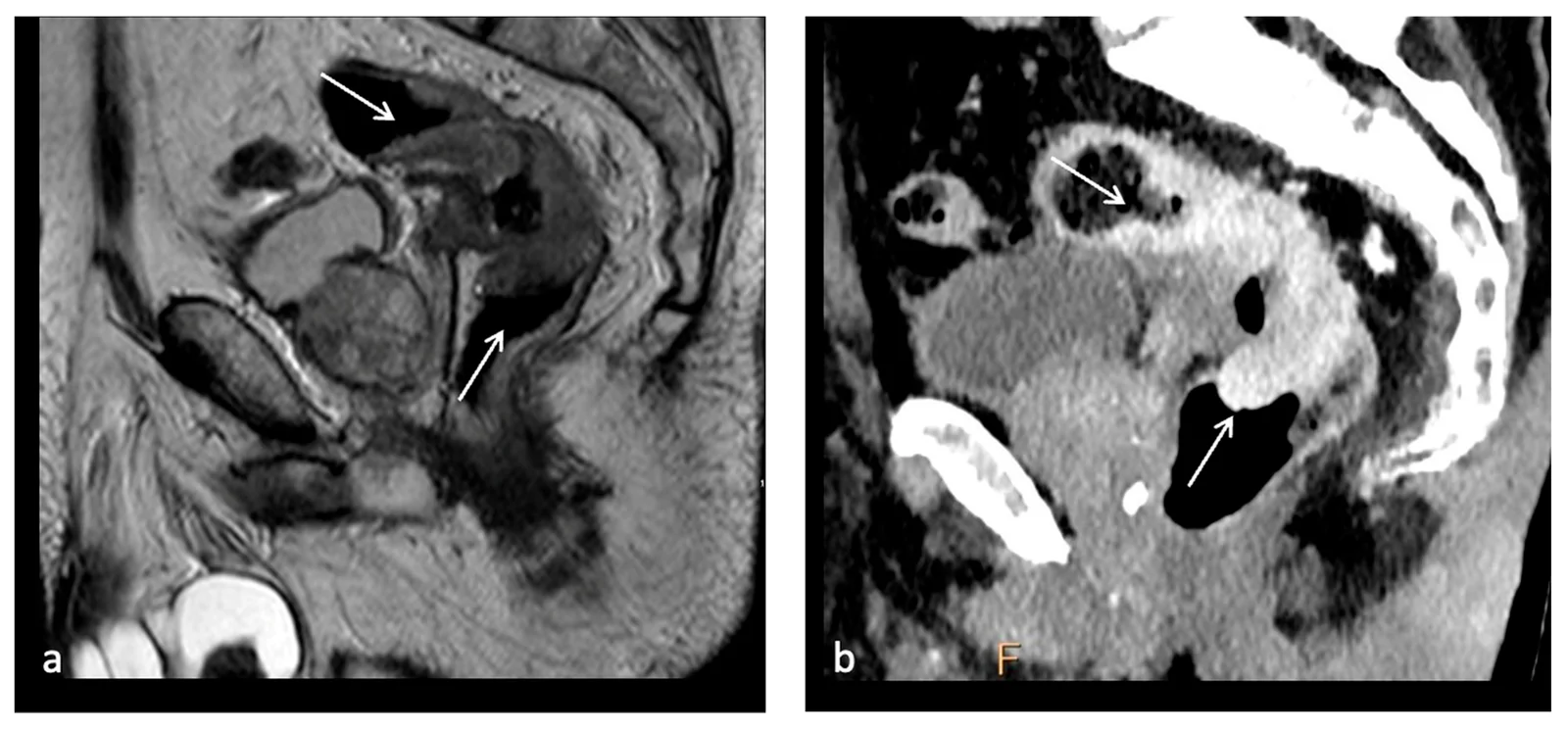
Concordant assessment of tumor location and longitudinal extent on MRI and dual-layer spectral CT. (**a**) Sagittal T2-weighted TSE MRI and (**b**) sagittal 40-keV virtual monoenergetic image reconstructed from the venous-phase dual-layer spectral CT dataset at the same anatomical level. The rectal tumor (arrows) is similarly depicted by both modalities, showing excellent concordance in the evaluation of lesion location and longitudinal extension.

**Figure 2:**
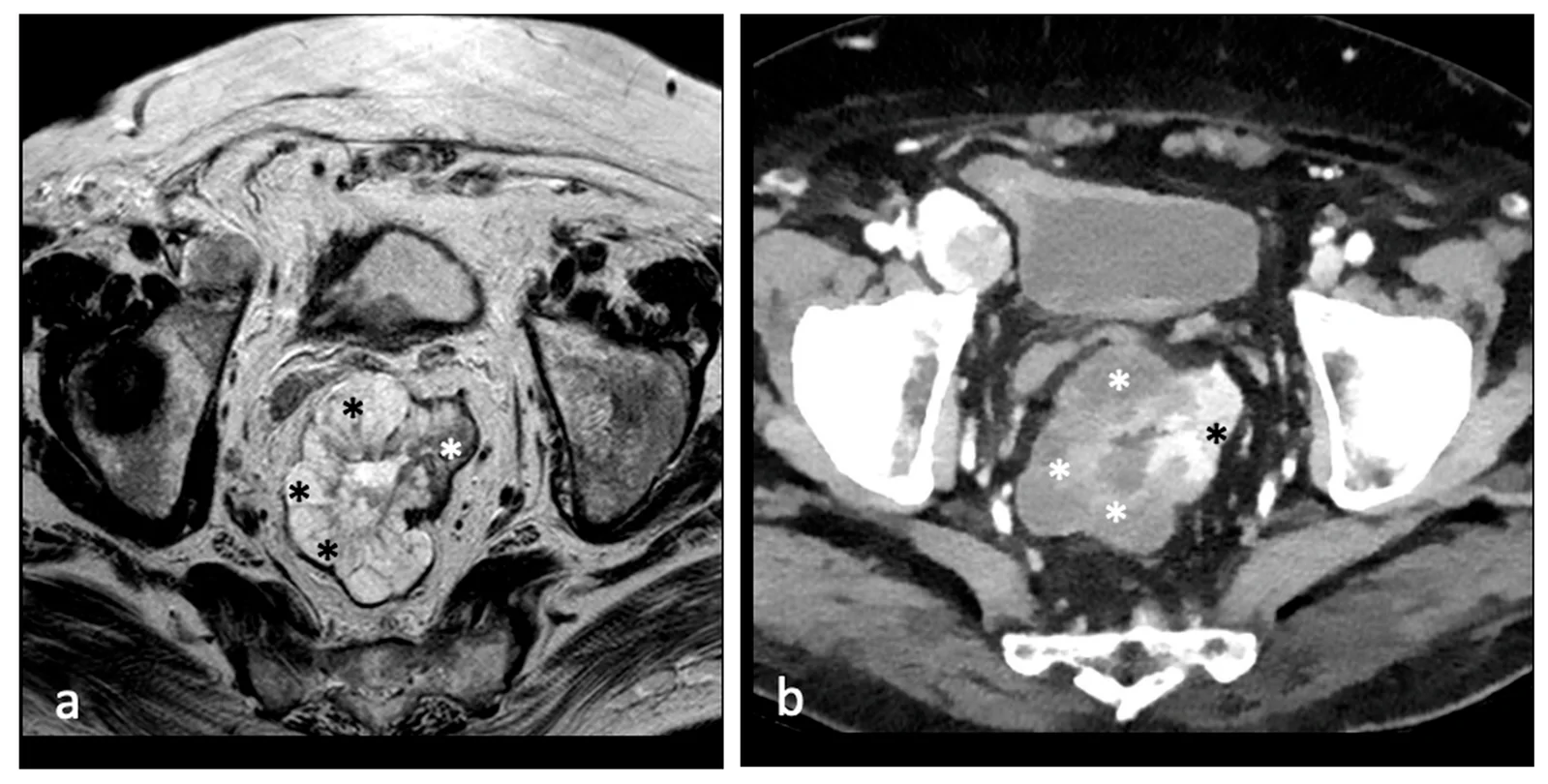
Concordant assessment of mesorectal infiltration in mucinous rectal adenocarcinoma on MRI and dual-layer spectral CT. (**a**) Axial T2-weighted turbo spin-echo (TSE) MRI and (**b**) axial 40-keV virtual monoenergetic image reconstructed from the venous-phase dual-layer spectral CT acquisition at the corresponding anatomical level. Both modalities demonstrate excellent agreement in the evaluation of mesorectal tumor extension. The mucinous component is characterized by marked hyperintensity on T2-weighted MRI (black asterisks) and low attenuation on CT (white asterisks), whereas the solid tumor component appears iso- to hypointense on MRI (white asterisk) and demonstrates increased enhancement on the contrast-enhanced spectral CT images (black asterisk).

**Figure 3:**
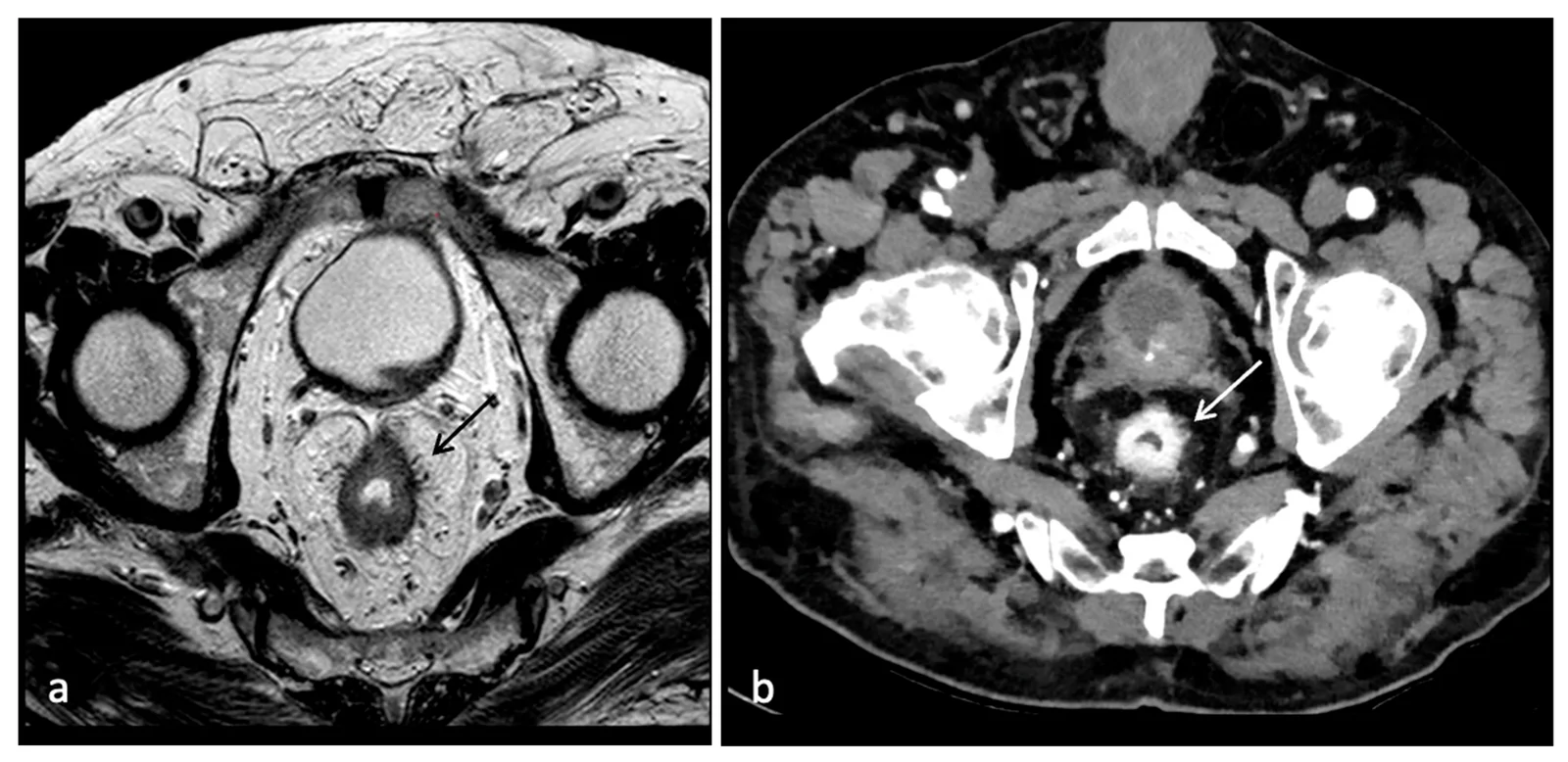
Concordant depiction of desmoplastic reaction on MRI and dual-layer spectral CT. (**a**) Axial T2-weighted turbo spin-echo (TSE) MRI and (**b**) axial 40-keV virtual monoenergetic image reconstructed from the venous-phase dual-layer spectral CT acquisition at the corresponding anatomical level. Both imaging modalities demonstrate spiculated desmoplastic strands extending from the primary rectal tumor into the surrounding mesorectal fat (arrow), showing excellent agreement in the depiction of peritumoral desmoplastic reaction.

**Figure 4:**
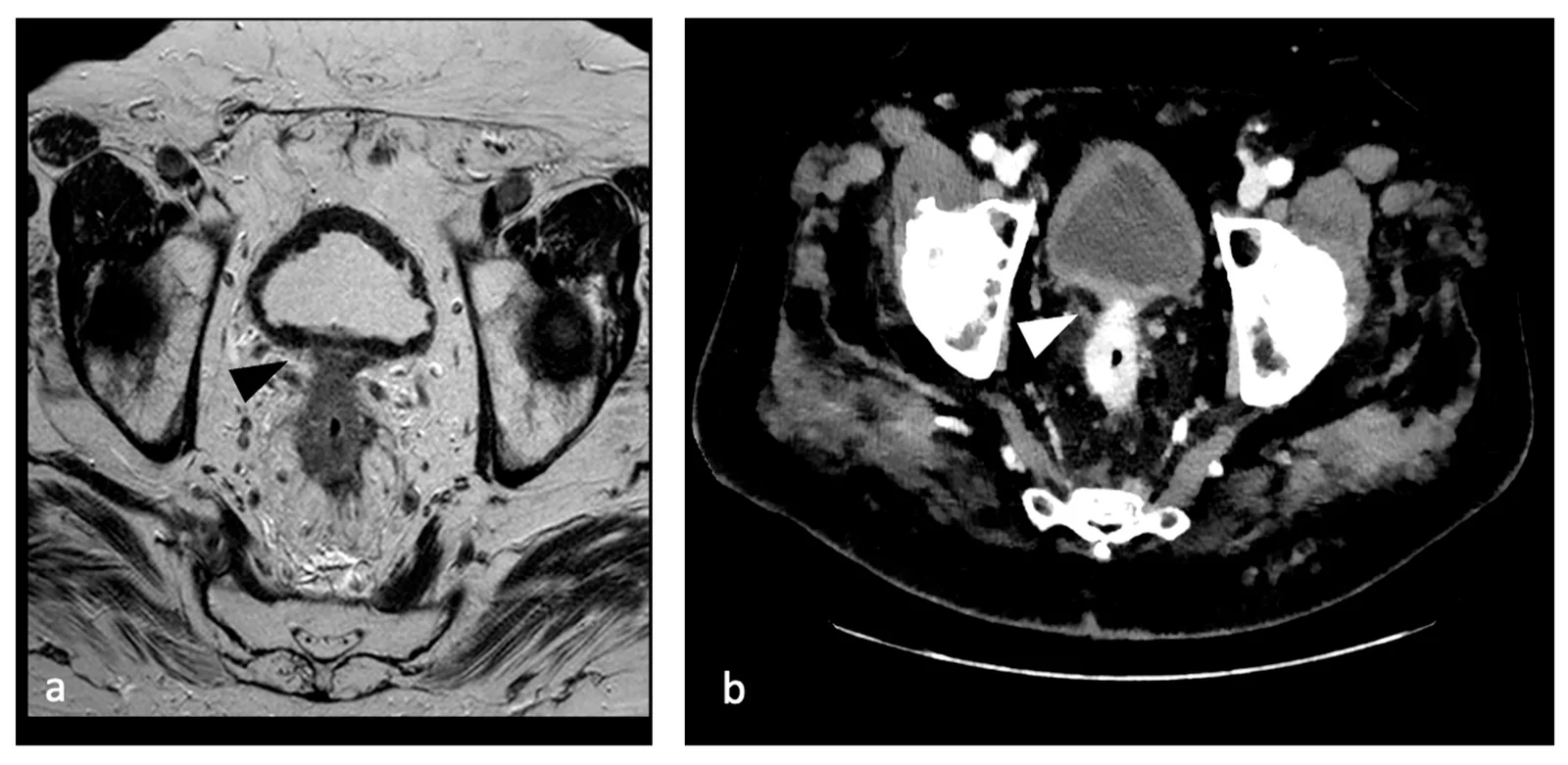
Concordant assessment of adjacent organ invasion on MRI and dual-layer spectral CT. (**a**) Axial T2-weighted turbo spin-echo (TSE) MRI and (**b**) axial 40-keV virtual monoenergetic image reconstructed from the venous-phase dual-layer spectral CT acquisition at the corresponding anatomical level. Both imaging modalities demonstrate direct tumor extension into the posterior wall of the urinary bladder (arrowhead), consistent with T4b disease, showing excellent agreement in the evaluation of locally advanced rectal cancer.

**Figure 5:**
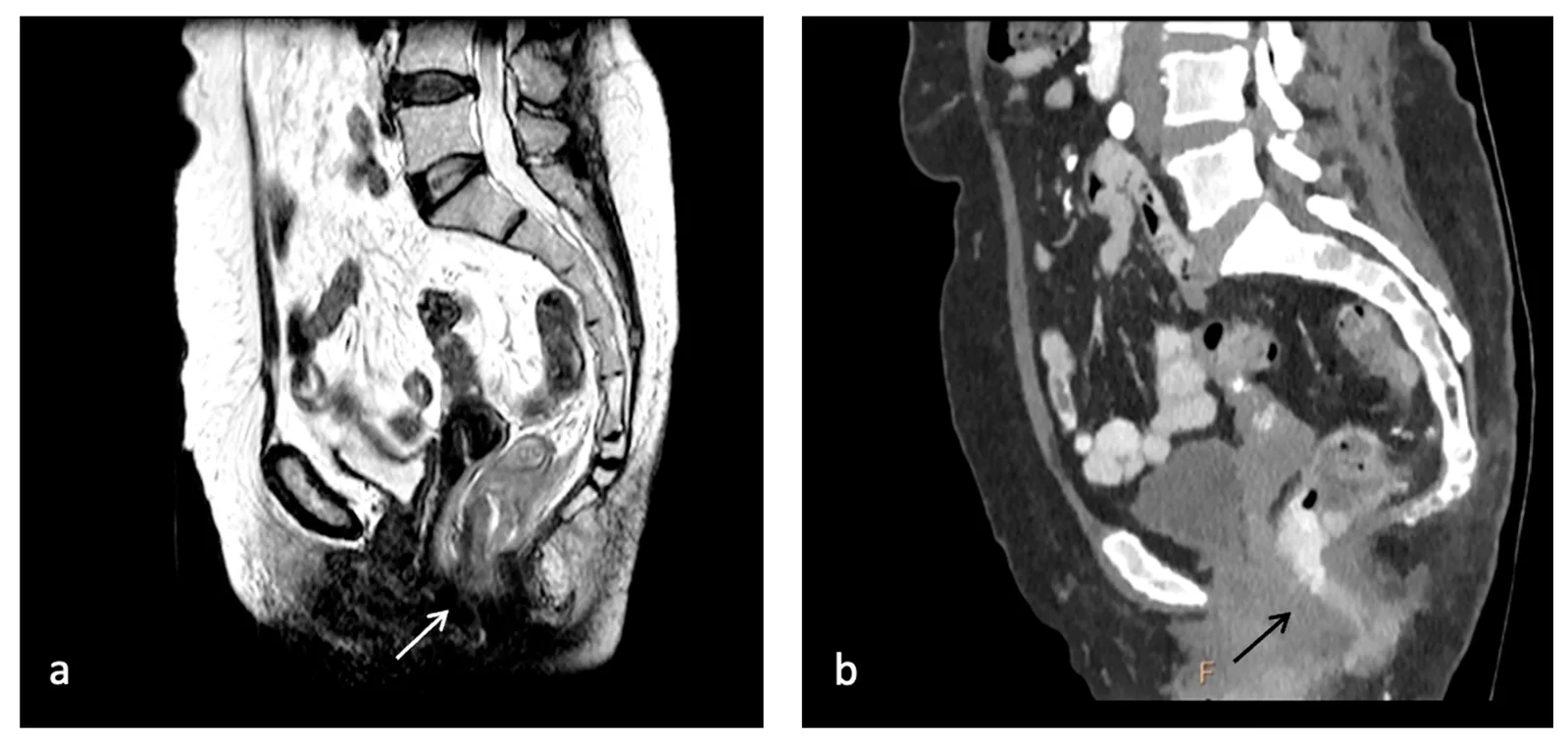
Concordant assessment of anal canal involvement on MRI and dual-layer spectral CT. (**a**) Sagittal T2-weighted turbo spin-echo (TSE) MRI and (**b**) sagittal 40-keV virtual monoenergetic multiplanar reconstruction obtained from the venous-phase dual-layer spectral CT acquisition. Both imaging modalities demonstrate tumor extension beyond the anorectal junction with direct involvement of the anal canal (arrow), showing excellent agreement in the evaluation of distal tumor spread.

No statistically significant differences were observed between MRI and DLSCT for any of the continuous measurements evaluated. Figure 1, Figure 2, Figure 3, Figure 4 and Figure 5 provide representative imaging examples.

The imaging examples illustrate findings that were subsequently evaluated in the quantitative analysis.

Median longitudinal tumor extent was 75.0 mm (IQR, 60.0–118.3) on MRI and 80.0 mm (IQR, 60.0–118.6) on DLSCT (*p* = 0.052). Median distance from the anorectal junction was 72.5 mm (IQR, 45.0–95.4) on MRI and 77.0 mm (IQR, 45.8–98.0) on DLSCT (*p* = 0.099). Median distance from the anal verge was 25.0 mm (IQR, 15.0–75.0) on MRI and 25.0 mm (IQR, 15.3–75.0) on DLSCT (*p* = 0.059). The paired comparisons are summarized in Table 2.

Complete agreement between DLSCT and MRI was observed for CRM involvement, anal canal involvement, peritoneal involvement, desmoplastic reaction, and suspicious lymph-node classification; for each feature, sensitivity, specificity, and accuracy were 100%, with κ = 1.00. For EMVI, DLSCT classified all 24 MRI-positive cases as positive but also classified two of seven MRI-negative cases as positive. This yielded 100% sensitivity, 71.4% specificity, 93.5% accuracy, and substantial agreement (κ = 0.79). Agreement for mesorectal infiltration was almost perfect (κ = 0.84; 95% CI, 0.64–1.00). Comparative results for the categorical staging features are presented in Table 3.

## 4. Discussion

MRI remains the established reference standard for locoregional staging of rectal cancer [6,7,9]. However, its limitations and the need for streamlined diagnostic pathways have encouraged investigation of complementary imaging strategies. In this preliminary study, venous-phase 40-keV DLSCT reconstructions showed high concordance with MRI for several locoregional imaging features, suggesting that spectral CT may provide information beyond its conventional role in distant staging [4,21,22].

From a practical standpoint, DLSCT offers the advantage of generating multiplanar reconstructions retrospectively from a single volumetric venous-phase acquisition, without the need for prospectively tailored imaging planes. This differs from rectal MRI, which requires dedicated high-resolution sequences oriented according to tumor location. Although workflow duration was not formally assessed in the present study, the standardized volumetric nature of DLSCT may facilitate image reconstruction and comparison across multiple anatomical planes. Detector-based DLSCT requires dedicated dual-layer detector hardware and specific spectral post-processing capabilities. Therefore, its availability remains dependent on local imaging infrastructure and may vary among institutions, which should be considered when assessing the generalizability of the present findings [13,15].

Economic considerations also deserve attention when comparing DLSCT with dedicated rectal MRI. A formal economic or cost-effectiveness analysis was not performed in the present study, and direct costs may vary substantially among institutions and healthcare systems according to equipment, software, maintenance, staffing, examination duration, and reimbursement policies. Detector-based DLSCT requires dedicated spectral CT hardware and post-processing capabilities. However, when DLSCT is already clinically indicated for systemic staging, the 40-keV virtual monoenergetic reconstructions used for locoregional assessment can be generated retrospectively from the same acquisition, without requiring an additional CT examination. The economic impact of such an approach would therefore depend not only on the cost of the individual examination but also on whether spectral information can reduce the need for an additional imaging session, as well as on local workflow and resource utilization. Formal cost-effectiveness studies comparing DLSCT-based and MRI-based diagnostic pathways would be valuable for healthcare providers and policymakers before any economic advantage of broader DLSCT implementation can be established.

A primary focus of this study was the assessment of local tumor extension, including CRM involvement, anal canal invasion, and peritoneal involvement. DLSCT showed complete concordance with MRI for these classifications. These results must be interpreted cautiously because of the small sample, the limited numbers of positive and negative observations, the consensus-reading design, and the use of MRI rather than histopathology as the reference. Nevertheless, the findings are consistent with previous work suggesting improved rectal tumor conspicuity on low-keV DLSCT reconstructions. Jia et al. reported favorable image-quality metrics and T-staging performance for 40-keV virtual monoenergetic images compared with high-resolution MRI [18].

EUS should also be considered within the multimodality approach to rectal cancer staging. Its principal strength lies in the detailed assessment of mural penetration, which is particularly relevant when differentiating early-stage tumors and selecting patients who may be candidates for local treatment. Kocaman et al., in a cohort of 50 patients with surgical pathology as the reference standard, reported higher T-staging accuracy for EUS than for MDCT and MRI, while nodal staging performance was comparable among the three techniques [12]. These findings underline the complementary role of EUS rather than its interchangeability with cross-sectional imaging. In contrast to MRI and DLSCT, EUS provides a more restricted assessment of the mesorectum and extramural structures and cannot simultaneously evaluate distant disease. EUS was not systematically performed in our cohort; consequently, a direct comparison with DLSCT could not be undertaken.

Dual-layer spectral CT simultaneously acquires spatially and temporally matched low- and high-energy information during a single examination, enabling retrospective reconstruction of virtual monoenergetic images. At low energy levels, iodine attenuation increases and may improve lesion-to-background contrast, although this benefit must be balanced against a potential increase in image noise. In the present study, 40-keV reconstructions were selected on the basis of previous evidence showing improved rectal tumor conspicuity and favorable image quality at this energy level [18]. The increased iodine-related contrast at low keV may have contributed to the observed morphological concordance with MRI; however, image appearance and noise characteristics are energy-dependent, and the present findings cannot necessarily be extrapolated to other monoenergetic levels, contrast phases, or spectral CT platforms. Importantly, low-keV CT imaging should not be considered to reproduce the tissue-contrast mechanisms of T2-weighted MRI, because the two modalities rely on fundamentally different physical principles. Qin et al. also reported significant differences in selected dual-energy CT parameters between T3 and T4a colorectal cancers, suggesting a potential role for quantitative spectral analysis in this challenging distinction [23].

One of the most prognostically relevant features in rectal cancer staging is extramural vascular invasion (EMVI) [24,25]. In our cohort, DLSCT reproduced all MRI-positive EMVI classifications, whereas specificity was 71.4%, with two additional DLSCT-positive findings among seven MRI-negative cases. The resulting agreement remained substantial (κ = 0.79). The lower specificity may reflect the difficulty of distinguishing vascular invasion from adjacent fibrosis, desmoplastic reaction, or other peritumoral changes on morphological imaging. Previous studies suggest that quantitative spectral biomarkers may provide additional information beyond visual assessment of low-keV images. Gao et al. reported high diagnostic performance of delayed-phase normalized iodine concentration for predicting histopathologically confirmed EMVI [26]. Zhang et al. and Chen et al. similarly found associations between spectral parameters and lymphovascular invasion [27,28]. The present analysis did not include iodine maps or other quantitative spectral parameters; their incremental value should be assessed in future studies.

The assessment of lymph-node involvement remains challenging in rectal cancer because size criteria alone are insufficient: small nodes may contain metastatic disease, whereas enlarged nodes may be reactive [6,9]. In our visual assessment, DLSCT showed complete concordance with MRI for the classification of suspicious lymph nodes. This finding should not be interpreted as confirmation of nodal metastasis, because histopathological nodal status was not used as the reference standard. Previous studies have nevertheless shown that quantitative dual-energy CT parameters, including normalized iodine-based indices and effective atomic number, may help distinguish metastatic from non-metastatic lymph nodes [29,30,31]. DECT-derived radiomic analysis has also shown promising results for nodal prediction, although these approaches remain investigational and require external validation [32].

Beyond morphological staging, quantitative spectral parameters may provide information associated with tumor aggressiveness. Previous studies have reported associations between iodine-based measurements or effective atomic number and pathological T stage, histological differentiation, mesorectal fascia involvement, and the distinction between advanced adenomas and early adenocarcinomas [33,34,35]. These applications were not evaluated in the present study and should therefore be regarded as potential directions for future research rather than established clinical uses.

The present study focused exclusively on visual assessment of 40-keV virtual monoenergetic images. Spectral CT also provides quantitative outputs, including iodine concentration, effective atomic number, and spectral attenuation characteristics, which may be combined with radiomic analysis. Future studies should determine whether these parameters provide reproducible incremental value for tumor characterization, treatment-response prediction, or prognostic stratification. Such applications remain investigational and require standardized acquisition, external validation, and correlation with histopathological and clinical outcomes.

Previous studies provide examples of how spectral CT findings can be integrated with histopathological data. Gao et al. correlated quantitative DECT iodine parameters with postoperative histopathological EMVI status in rectal cancer [26], while Chen et al. evaluated associations between DLSCT-derived quantitative parameters and pathological features including tumor differentiation and lymphovascular invasion [28]. Other investigators have correlated spectral CT parameters with pathological T stage and histological grade [34]. At the nodal level, Al-Najami et al. performed dual-energy CT of rectal cancer surgical specimens and used histopathological evaluation of matched mesorectal lymph nodes as the reference standard [29]. These approaches provide a methodological framework for future DLSCT studies, in which preoperative spectral imaging findings could be systematically matched with surgical histopathology at the primary tumor and, whenever feasible, at the nodal level. Such correlation would allow diagnostic performance against pathology to be distinguished from imaging concordance with MRI.

Several limitations should be acknowledged. First, MRI rather than histopathology was used as the reference standard. Although MRI represents the established imaging standard for locoregional staging, it remains imperfect for specific tasks, including differentiation of limited mural from early extramural disease, characterization of small mesorectal lymph nodes, and assessment of some peritumoral vascular or fibrotic changes [6,7,8,9,10,11]. Consequently, any MRI misclassification may be propagated into the comparative metrics reported in this study. DLSCT findings classified as false-positive or false-negative relative to MRI cannot therefore be assumed to represent true diagnostic errors, and complete concordance between the two modalities does not necessarily imply agreement with histopathology. The absence of systematic histopathological correlation prevents determination of which modality was correct in discordant cases and precludes definitive assessment of DLSCT accuracy for microscopic tumor extension, EMVI, nodal metastasis, and other pathological features. Second, the retrospective, single-center design and small sample size limit the generalizability of the findings and produce wide confidence intervals, particularly for categorical features represented by few positive or negative cases. Moreover, because inclusion required the availability of both pretreatment MRI and DLSCT, selection bias cannot be excluded, and the study population may not fully represent the broader population of patients undergoing staging for rectal cancer. Third, all readings were performed by consensus; therefore, inter-reader reproducibility could not be assessed. Fourth, the analysis was restricted to visual evaluation of 40-keV virtual monoenergetic images and did not include iodine maps or other quantitative spectral outputs. Finally, no external validation cohort was available. Future validation should be pursued through prospective multicenter studies with consecutive patient enrollment, predefined eligibility criteria, prespecified endpoints, and an a priori sample-size calculation. MRI and DLSCT should be interpreted independently by blinded readers, with inter-reader reproducibility formally assessed and surgical histopathology used as the reference standard whenever feasible for local tumor extension, EMVI, and nodal status. Future studies should also determine whether quantitative spectral parameters provide incremental value over visual 40-keV assessment before DLSCT can be considered for a broader role in routine locoregional staging.

## 5. Conclusions

In this preliminary single-center study, DLSCT using 40-keV virtual monoenergetic images showed high concordance with MRI for several locoregional imaging features of rectal cancer. These findings support a potential complementary role for DLSCT, particularly when MRI is contraindicated or inconclusive. Because MRI was used as the imaging reference standard and systematic histopathological validation was unavailable, the results should not be interpreted as evidence that DLSCT can replace dedicated rectal MRI.

Larger prospective multicenter studies with pathological correlation are needed to validate these findings across more diverse patient populations, improve their generalizability, and better define the diagnostic role of DLSCT and its potential contribution to integrated local and systemic staging.

## Figures and Tables

**Table 1:** Patient demographic characteristics.

Variable	Value
Number of patients	31
Male, *n* (%)	21 (67.7%)
Female, *n* (%)	10 (32.3%)
Mean age (years)	69 ± 13.3

**Table 2:** Comparison of continuous measurements between MRI and DLSCT.

Parameter	*p*-Value	DLSCT Median [IQR]	MRI Median [IQR]
Longitudinal tumor extent	0.052	80.0 [60.0–118.6]	75.0 [60.0–118.3]
Anorectal junction distance	0.099	77.0 [45.8–98.0]	72.5 [45.0–95.4]
Anal verge distance	0.059	25.0 [15.3–75.0]	25.0 [15.0–75.0]

**Table 3:** Concordance of DLSCT with MRI for categorical staging features.

Parameter	MRI ±, *n*	Sensitivity (95% CI)	Specificity (95% CI)	Accuracy	κ
CRM involvement	17/14	100% (80.5–100.0)	100% (76.8–100.0)	100%	1.00
Anal canal involvement	18/13	100% (81.5–100.0)	100% (75.3–100.0)	100%	1.00
Peritoneal involvement	10/21	100% (69.2–100.0)	100% (83.9–100.0)	100%	1.00
EMVI	24/7	100% (85.8–100.0)	71.4% (29.0–96.3)	93.50%	0.79
Desmoplastic reaction	25/6	100% (86.3–100.0)	100% (54.1–100.0)	100%	1.00
Suspicious lymph nodes	20/11	100% (83.2–100.0)	100% (71.5–100.0)	100%	1.00

Note: MRI was used as the imaging reference standard. MRI ± indicates the numbers of MRI-positive and MRI-negative cases. Accuracy represents the proportion of concordant MRI and DLSCT classifications. κ, Cohen’s kappa; EMVI, extramural vascular invasion.

## Data Availability

The data supporting the findings of this study are available from the corresponding author upon reasonable request. The data are not publicly available because they contain information that could compromise patient privacy.

## Abbreviations

The following abbreviations are used in this manuscript:

CI	Confidence Interval
CRM	Circumferential Resection Margin
CT	Computed Tomography
DECT	Dual-Energy Computed Tomography
DLSCT	Dual-Layer Spectral Computed Tomography
DWI	Diffusion-Weighted Imaging
EMVI	Extramural Vascular Invasion
IQR	Interquartile Range
kVp	Kilovolt Peak
MRI	Magnetic Resonance Imaging
SD	Standard Deviation
TNM	Tumor–Node–Metastasis
TSE	Turbo Spin Echo
